# Rational catering of irrational emotions: Investor sentiment and executive tone

**DOI:** 10.3389/fpsyg.2022.987310

**Published:** 2022-09-13

**Authors:** Jing Qiu, Ni Yang

**Affiliations:** School of Accounting, Guizhou University of Finance and Economics, Guiyang, China

**Keywords:** investor sentiment, executive tone, the Catering Theory, incentive, expectation, gambling tendency

## Abstract

In China, investors generally make decisions depending on the intonation of executive announcements. A total of 20,328 observations are sampled from the Chinese equity market between 2005 and 2019. We perform principal component analysis to produce monthly sentiment indices and calculate the weighted average of the value over the fiscal year to measure the degree of investor sentiment. The results of the empirical analysis reveal that: (1) there is a significant positive correlation between market-level investor sentiment and executive tone, (2) stock price and trading-volume pressures on executives, and firm-level investor expectation and gambling tendency positively moderate the relationship between investor sentiment and executive tone, (3) executive optimism does not mediate the association between investor sentiment and executive tone, and (4) manipulated and real intonations are non-homogeneous tactics adopted by rational managers to cater to changes in investor sentiment. These findings indicate that executives' intonations are both an extension of firms' current and past performances and managers' decision-making based on sentiments and behaviors of irrational investors, which are consistent with the Catering Theory. In addition, rational executives tend to adopt various intonation tactics to respond to investor sentiment to avoid declines in stock prices and trading volumes.

## Introduction

The efficient market hypothesis is an investment theory refined and developed by Fama (1965), which states that asset prices reflect all market information. However, many researchers have attacked this hypothesis by proposing financial market phenomena such as the price-earnings ratio anomaly, herding effects, and noisy trading (Friend et al., 1970; De Bondt and Thaler, 1985; Kyle, 1985). They also believe that it is hard to verify the efficient market hypothesis in practice since investors are constantly irrational.

The traditional financial theory assumes that stock investors are rational, and stock prices reflect firms' values (Fama, 1965). However, some scholars argue that investors are completely irrational, and they trade based on the company's information disclosures in the past and present (Kahneman and Tversky, 1979). Investor sentiments are the primary factors of capital market mispricing, according to the Behavioral Finance Theory (Baker and Wurgler, 2006; Stambaugh et al., 2012; Huang et al., 2015) especially in the Chinese capital market. On October 8, 2018, some negative market information largely influenced Chinese capital market investors, making investors sell equities. That caused the Shanghai Stock Exchange index to drop by 3.72% within 24 h. As seen, the research related to investor sentiment is theoretically and practically relevant.

In China, executives tend to make decisions based on investors' professional characteristics and judgment, as these criteria influence stock prices. According to some experts, investor sentiment influences firms' investment decisions and financing behaviors (Elbannan, 2020; Byun et al., 2021). Executives raise dividend payments in response to the positive sentiments of investors, referring to (Baker and Wurgler, 2004a) Catering Theory. Some studies have also developed the Catering Theory, revealing that companies take financing actions as investments (Polk and Sapienza, 2009), name changes (Cooper et al., 2005), and earnings management (Simpson, 2013; Kong, 2018) to cater to investor sentiment. As a high-context country, Chinese capital market investors prefer easy-to-understand narrative information, especially intonation information (Li, 2010; Huang et al., 2014). This study examines whether investors' irrational sentiments influence the tone of executives.

Our research contributes to a variety of previous studies. First, we provide a contribution to research regarding the determinants of executive tones. Huang et al. (2014) find that managers change their intonations primarily based on company performance, earnings, risk, and complexity. Our findings regarding the impact of investor sentiment on executive tone provide another channel for understanding executive motivations to manage disclosure tones.

Second, this paper provides significant support for the Catering Theory from a new perspective—that of executive intonations. Previous literature verifies the Catering Theory in the contexts of cash dividends (Baker and Wurgler, 2004b), name changes (Cooper et al., 2005), and earnings management (Kong, 2018). However, we find that executives also employ and even manipulate intonation tactics in response to changes in investor sentiment. These findings add to the body of knowledge by extending previous research regarding the specific catering activity methods.

Third, our findings differ from Baker and Wurgler (2004b), Chazi et al. (2018), and Kong (2018), all of whom verify the Catering Theory from the perspectives of either market-wide investor sentiment or firm-level investor behavior. By contrast, we investigate how executive tone is affected by investor sentiment at the market level and how investor behavior at the firm level affects the relationship between these two factors; we thereby complement the findings of previous studies.

Finally, few studies have explored the executive motivations for engaging in activities that cater to investor sentiment. In this study, we use moderating analysis to examine the potential catering motivations of managers, finding that executives adopt positive intonations in response to investor sentiment to avoid declines in stock prices and trading volumes. These findings extend Catering Theory research from the perspective of catering incentives.

## Literature review and hypotheses

### Literature review

Previous studies argue that investor sentiment deviates from rational expectations (Barberis et al., 1998; Ruan et al., 2020). According to Chue et al. (2019), investor sentiment represents a misestimate of asset values by stockholders. Investor sentiment is also characterized by speculative tendencies and inaccurate assessments of asset prices, according to Baker and Wurgler (2007). The development of the Behavioral Finance Theory has led researchers to pay attention to the impact of investor sentiment on business decision-making. Generally, studies take one of three routes to explain how investor emotions affect business performance. The first is the equity-financing route, where investor sentiment affects corporate actions by expanding financing resources (Chang et al., 2007). The second is the emotional contagion route, where investor sentiment affects executives' optimistic expectations and influences their decisions (Hirshleifer and Teoh, 2009). The third is the catering route, where executives make financial decisions to meet the investors' expectations (Kong, 2018; Elbannan, 2020). Some studies show that managers change their dividends (Chazi et al., 2018), corporate names (Cooper et al., 2005), and investment choices (Polk and Sapienza, 2009) to cater to irrational investors. Meanwhile, other researchers argue that managers adopt information disclosures, such as earnings management, to respond to investor sentiment (Simpson, 2013; Kong, 2018). However, few studies explore the impact of investor sentiment on manager narrative disclosures, especially the impact on executive tone.

The development of computer language analysis technology in recent years has led an increasing number of researchers to focus on narrative information, especially executive tone (Bowen et al., 2018; Castro et al., 2019). These researchers argue that as a supplement to data information, narrative disclosures are an important means of acquiring additional information about company performance and earnings (Kothari et al., 2009; Li, 2010). Therefore, this study's focus on the determinants of executive tone places it at the cutting edge of economics research. In addition, previous studies find that managers with information advantages can change the tones of their disclosures to achieve personal goals, including managing impressions, demonstrating the reasonableness of compensation, and reducing litigation risk (Huang et al., 2014; Arslan-Ayaydin et al., 2016; Luo and Zhou, 2019). Huang et al. (2014) and Hossain et al. (2020) argue that executives even can manipulate the tones of their narrative disclosures. This paper examines how executive tone can be strategically controlled or manipulated to cater to investor sentiments and behaviors, extending the related literature on information manipulations. In practice, since Chinese is a complex language system (Tan et al., 2014; Davis et al., 2015) and our research verifies the Catering Theory of executive tones using a sample from the Chinese stock market, our findings have practical implications for regulators and corporate actors in China and other high-context countries.

### Hypotheses

Developed by Baker and Wurgler (2004a,b), the Catering Theory was originally used to explain the phenomenon of companies making financial decisions to satisfy investor needs and preferences. Investor sentiment is the principal reflection of investor preferences and emotional needs, which means executives are incentivized to take actions that cater to it. On the one hand, since companies and investors demand and supply capital in the equity market, respectively, rational managers tend to respond to investor sentiment to satisfy the needs and preferences of capital suppliers (Baker and Wurgler, 2004b). On the other hand, the equity market comprises many irrational investors, whose sentiments significantly influence stock transactions and prices (Hong and Stein, 2003). Therefore, rational executives are intensely incentivized to respond to rising investor sentiment to avoid firm earnings and performance that disappoint investors and, by extension, result in negative consequences, such as stock trading and price declines (Chazi et al., 2018).

Of course, managers also have the ability to assess and cater to investor sentiment. Executives with information and experience advantages can determine whether investor sentiment is optimistic based on their expectations and behaviors as reflected in equity trading or other situations (Bilel and Mondher, 2021). Meanwhile, prior research reveals that executives can engage in financial activities that cater to investor sentiments, such as cash dividends (Baker and Wurgler, 2004a), investments (Polk and Sapienza, 2009), and name changes (Cooper et al., 2005). Thus, we deduce that as companies' decision-makers, managers can take various actions and deploy effective tactics in response to investors' sentiments if they want.

Executive tone is an extension of enterprise earnings and performance. In reality, positive intonation simply means that executives adopt more positive words relative to negative words in their narrative disclosures (Loughran and McDonald, 2016), which signals that executive sentiment and expectations regarding future corporate earnings are optimistic. Therefore, managers can employ positive tones to cater to investor sentiment by conveying positive signals about future earnings and values. In addition, the input and supervision costs of catering intonation are lower than the those of traditional catering methods (Simpson, 2013; Huang et al., 2014). Managers thus tend to change their tones to satisfy investor needs and cater to investor sentiment rather than employing traditional catering tactics. Nevertheless, the question of how managers change their tones to cater to investor sentiment remains. According to Mian and Sankaraguruswamy (2012), the demand for good news among optimistic investors is intense, while pessimistic investors dislike bad news. We thus believe that rational managers tend to adopt more positive tones in response to over-optimistic investor sentiment and behavior. However, when irrational investor sentiment declines, executives with lower catering motives tend to deploy fewer positive intonation tactics—e.g., reducing numbers of positive words relative to negative words or engaging in less tone manipulation. Consequently, we propose the following hypothesis.

H1: The executive tone is positively correlated with market-level investor sentiment.

According to the Behavioral Finance Theory, personal incentives tend to drive executive behaviors and decisions. As discussed previously, executives cater to investor sentiment to avoid short-term stock price and trading volume declines (Baker et al., 2012). Thus, we deduce that higher stock price and trading-volume pressures foster stronger catering motivations in executives, leading them to employ more positive tones in their disclosures, conveying optimistic expectations and sentiments to avoid investor disappointment in firm earnings and value as well as declines in trading volumes and share prices. Thus, we propose the following hypotheses:

H2a: Stock price pressure on executives positively moderates the impact of investor sentiment on executive tone.
H2b: Stock trading-volume pressure on executives positively moderates the impact of investor sentiment on executive tone.

Rational executives assess irrational investor sentiment based on their trading behaviors in the capital market as well as their company-related expectations and preferences (Kong, 2018). Among them, positive investor expectations reflect their belief in high returns (Chazi et al., 2018), while gambling tendencies reveal their preferences for seeking higher returns with lower investments (Kumar, 2009). Thus, investors' expectations and gambling tendencies can influence their attitudes toward and choices regarding stock trading (Statman, 2002; Kumar, 2009) and affect corporate value and stock performance indirectly. Correspondingly, managers may make decisions, such as catering activities based on firm-level investor expectations and gambling tendencies. On the other hand, the rapid development of the Internet and technology provide adequate information about stock trading and market evaluations for the managers, who have the advantages of information and governance to effectively detect and determine investors' optimistic expectations and gambling tendencies in stock trading (Bilel and Mondher, 2021). In addition, investors with optimistic expectations and higher gambling tendencies may overestimate or underestimate the probabilities of gains or losses, which leads them to increase investments and stock purchases after receiving positive information (Miller, 1977; Morellec and Schurhoff, 2011). As a result, managers with the incentives of avoiding declines in share prices and trading volumes tend to cater to increasing investor expectations and gambling tendencies to achieve these goals. That promotes rational executives to adopt more positive tones to cater to rising investor sentiment when investors' expectations are over-optimistic and gambling tendencies are higher. We thus propose the following hypotheses:

H3a: Firm-level investor expectation positively moderates the impact of investor sentiment on executive tone.
H3b: Firm-level investor gambling tendency positively moderates the impact of investor sentiment on executive tone.

## Materials and methods

### Sample and data

Considering that content and format guidelines for annual reports revised by the China Securities Regulatory Commission (CSRC) in 2005 specified the content is required to be included in MD & A, we thus select a sample of publicly traded companies from 2005 to 2019 from the China Stock Market & Accounting Research Database (CSMAR) and the Chinese Research Data Services Database (CNRDS). Meanwhile, we use Python software to acquire annual reports from the Shanghai Exchange and Shenzhen Exchange to extract the management analysis and discussion sections for textual analysis. In addition, the samples are selected using several criteria: (1) we exclude the samples from banking, insurance, and other financial industries, (2) we delete the observations with ST (special treatment) and PT (particular transfer) firms' transaction status, (3) we exclude observations whose liabilities are more than assets, and (4) we delete the observations with unavailable data. Finally, we obtain 20,328 firm-year observations and winsorize the top and bottom 1% of each variable to reduce the impact of extreme observations.

### Variable definitions

#### Executive tone

We use textual analysis to measure executive intonation following the Loughran and McDonald (2016) approach and extract the management analysis and discussion texts from annual reports to delete stop words. Based on the dictionaries generated using HOWNET, DLUTSD, and NTUSD, we calculate the numbers of positive and negative words in each text using Python software. We use Equation (1) to calculate the executive tone variable (*Tone*), and a higher value indicates a more positive management intonation.

(1) $$\begin{array}{l} Tone_{i,t}=\left(Postsum_{i,t}-Negsum_{i,t}\right)/\left(Postsum_{i,t}+Negsum_{i,t}\right) \end{array}$$

where, *Postsum* and *Negsum* are the numbers of positive and negative words, respectively.

#### Investor sentiment

Based on the approach adopted by Baker and Wurgler (2006), this study uses principal component analysis to construct the investor sentiment variable (*Sentiment*). We use 10 indicators, including five proxy variables shown in Table 1 and their lagged one-period variables to perform a principal component analysis. We then obtain a weighted average of the analysis as the initial indicator of investor sentiment. Second, we perform a correlation analysis of the 10 indicators and the initial sentiment indicator to choose the variables with the highest correlation between the current and lagged period variables. Third, we construct the regression of three macroeconomic variables (the consumer price index, the industrial output price index, and the macroeconomic prosperity index) with each of the five selected indicators, to obtain the five residuals as the sentiment indicators excluding macroeconomic effects. Finally, we perform a principal component analysis of the five residuals to produce a weighted average of a monthly index and calculate the arithmetic average of the monthly index over the fiscal year to measure the investor sentiment (*Sentiment*). A higher value represents that market-wide investor sentiment is more positive.

**Table 1:** Proxy variables for investor sentiment.

**Variables**	**Name**	**Definition**
The discount rate of closed-end funds	*Cefd*	The weighted average discount rate of the closed-end funds' market value on the last trading day of the month.
The number of IPOs	*Ipon*	The number of initial public companies of the month.
The yield of IPO first day	*Ipor*	The arithmetic average of the newly listed A-shares' first-day returns of the month.
The number of new A-share accounts	*Niac*	The number of new accounts opened by investors of the month.
Turnover rate	*Turn*	The weighted average turnover rate of the outstanding stocks' market value of the month.

We adopt market-level investor sentiment as the independent variable for two main reasons. The first is that this paper makes efforts to examine how executive tone caters to irrational investors from a comprehensive perspective, we thus calculate the market-level investor variable (*Sentiment*) and the firm-level investor variables (*Expectation* and *Gamble*) for empirical analysis. The second is that we adopt the variable of market-level investor sentiment (*Sentiment*) that can alleviate the quid endogeneity between executive tone and investor sentiment because the impact of corporate executive intonation on market-level investor sentiment is relatively limited.

#### Executive pressure (stock-price pressure and trading-volume pressure)

Based on the risk-aversion assumption, potential risk and uncertainty may increase executives' disgust and stressful emotions and even influence their decision-making (Sauner-Leroy, 2004). Therefore, we argue that higher volatilities in stock trading prices and volumes in the short-term increase executives' perception of higher uncertainty risk and exert pressure on managers.

Referring to the approach adopted by Henry (2006), we use the event research method to construct the variables. We select the date of annual report disclosure as the benchmark date. And then, we calculate the standard deviations of stock price daily returns and daily turnover rates for the 30 days before the benchmark date as the variables of stock-price pressure (*Pricepressure*) and trading-volume pressure (*Volumepressure*), to accurately measure the degrees of stock price and trading volume pressures on executives before annual report disclosures, respectively.

#### Investor expectation

With rapid information technology and Internet development, managers can access information released by investors from forums directly. In addition, the investors' comments on the forums reflect their expectations and appraisals and can be easily detected and distinguished by managers. Therefore, we acquire the investors' comment data from the CNRDS database and then use Equation (2) to calculate the variable of investor expectation (*Expectation*) referring to the methods adopted by Tetlock et al. (2008) and Loughran and McDonald (2016). It is important to note that the CNRDS database has categorized investors' forum comments into positive, negative, and neutral comments based on machine learning methods.

(2) $$\begin{array}{l} Expectations_{i,t}\;=\\ \frac{\left(Positive\text{\_}comments\;-\;negative\text{\_}comments\right)}{Total\text{\_}comments} \end{array}$$

where, *Positive_comments, Negative_comments*, and *Total_comments* are the numbers of positive, negative, and total comments on forums, respectively.

#### Investor gambling tendency

Considering that gambling investors prefer stocks with high trait volatility, high trait skewness, and low prices (Kumar, 2009), we measure the degree of investor gambling tendency, referring to the method adopted by Kumar et al. (2016). The low price reflects the gambling tendency of pursuing big gains with small costs, high trait skewness implies a small probability of extremely high returns, and high trait volatility indicates a high risk for investment. Therefore, we calculate three indicators, including stock price (*Price*), volatility (*Ivol*), and skewness (*Iskew*) to find out the stocks in which actual and potential investors have higher gambling tendencies.

We calculate the ratio of monthly turnover prices of the stocks to the total shares to measure the stock price (*Price*). We then construct Equation (3) referring to the method adopted by Ang et al. (2006) and calculate the residual of the regression as the measurement of abnormal excess rate of return (*Abs_return*):

(3) $$\begin{array}{l} r_{i,d}-r_{f,d}\;=\;\alpha_{i,d}\;+\;\beta_{MKT,d}\left(MKT_{d}\;-\;r_{f,d}\right)\;+\\ \beta_{SMB,d}SMB_{d}\;+\;\beta_{HML,d}HML_{d}\;+\;\varepsilon_{i,d} \end{array}$$

where, *r*_*i,d*_ is the stock *i's* return rate on the day d of the month; *r*_*f,d*_ is the stock *i's* risk-free rate of return on the day d of the month; *MKT*_*d*_ − *r*_*f,d*_ is the market risk premium on the day d of the month; *SMB*_*d*_ is the simulated portfolio returns for the market size factor on day d of the month; *HML*_*d*_ is the simulated portfolio returns for the market book-to-market factor on the day d of the month.

We use Equations (4) and (5) to calculate the stock *i's* idiosyncratic volatility (*Ivol*) and idiosyncratic skewness (*Iskew*) of the month *t*.

(4) $$\begin{array}{l} Ivol_{i,t}\;=\;{\left[\frac{1}{N_{i}\left(t\right)}\Sigma_{d\in S_{i}\left(t\right)}{Abs\text{\_}return}_{i,d}^{2}\right]}^{\frac{1}{2}} \end{array}$$

(5) $$\begin{array}{l} Iskew_{i,t}\;=\;\frac{1}{N_{i}\left(t\right)}*\frac{\Sigma_{d\in S_{i}\left(t\right)}{Abs\text{\_}return}_{i,d}^{3}}{Ivol_{i,t}^{3}} \end{array}$$

where, *S*_*i*_(*t*) is the stock *i's* set of trading days of the month *t*; *N*_*i*_(*t*) is the stock *i's* number of trading days of the month *t*.

Investors who purchase or pay attention to gambling stocks with high trait volatility, high trait skewness, and low prices are more likely to be gambling investors. We thus adopt the sorting method to measure the gambling tendency of each stock's investors. In each month, the stocks are sorted depending on their idiosyncratic volatility, stock average price, and idiosyncratic skewness. We sort the idiosyncratic volatility and idiosyncratic skewness from the lowest to the highest, and the stock average price from the highest to the lowest.

Finally, we obtain the monthly gambling indices from Equation (6) and calculate the arithmetic average of the value over the fiscal year as a measurement of investor gambling tendency (*Gamble*). A larger value of *Gamble* means that the stock's actual and potential investors are more likely to be gambling investors (Kumar et al., 2016).

(6) $$\begin{array}{l} Gamble_{i,t}\;=\\ \left(\frac{{Ivol\text{\_}rank}_{i,t}}{N}\;+\;\frac{{Iskew\text{\_}rank}_{i,t}}{N}\;+\;\frac{{Price\text{\_}rank}_{i,t}}{N}\right)/3 \end{array}$$

where, *Ivol*_*rank*_*i,t*_ is the stock *i's* idiosyncratic volatility ranking of the month *t*; *Iskew*_*rank*_*i, t* is the stock *i's* idiosyncratic skewness ranking of the month *t*; *Price*_*rank*_*i,t*_ is the stock *i's* average price ranking of the month *t*; *N* is the number of stocks.

#### Control variables

Following prior research (Huang et al., 2014; DeBoskey et al., 2019; Hossain et al., 2020; Liu and Nguyen, 2020), we select determinants to control the factors that affect the tone of executives. Considering that executives' narrative disclosures are based on company performance and earnings, we include five earnings variables (*Roa, Roa_sd, Droa, Froa*, and *Loss*) and five performance variables (*Lev, Size, Growth, Cashflow*, and *Age*) (Huang et al., 2014). To capture factors about companies' value and stakeholders that could influence executive tone (DeBoskey et al., 2019; Hossain et al., 2020), we also include three value variables (*Btm, Ret*, and *Ret_sd*) and three stakeholders variables (*Occupy, Big4*, and *Mshare*). Moreover, given that the length of the report plays an important role in influencing the executive tone (Liu and Nguyen, 2020), we include the text-length variable (*Length*) to control the impact of text features. Table 2 reports the variable definitions.

**Table 2:** Variable definitions.

**Variables**	**Definition**
*Tone*	For each text of management analysis and discussion, we calculate the executive intonation variable using Equation (1).
*Sentiment*	We utilize the principal component analysis of five selected investor indicators to calculate the investor sentiment variable.
*Pricepressure*	The standard deviation of stock price daily returns for the 30 days before the benchmark date.
*Volumepressure*	The standard deviation of daily turnover rates for the 30 days before the benchmark date.
*Expectation*	We calculate the variable of *Expectation* using Equation (2).
*Gamble*	We calculate the monthly gambling indices using Equation (6) and use the arithmetic average of the value over the fiscal year as the variable of the investor gambling tendency.
*Overpositive*	The dummy variable takes to be 1, if the actual company profit level is lower than the estimated earnings level at least once in the sample period in 1Q, 6Q, 3Q, and yearly reports.
*Manitone*	The residual of Equation (13).
*Realtone*	The fitted value of Equation (13).
*Roa*	Returns on assets.
*Lev*	Debts on assets.
*Size*	The logarithm of the total assets.
*Growth*	The sales revenue growth rate.
*Cashflow*	Net cash flows on assets.
*Age*	The logarithm of the years of listing.
*Loss*	A dummy variable which takes to be 1 when *Roa* is negative.
*Btm*	Book value on market value.
*Ret*	Contemporaneous annual stock returns are calculated using monthly return data.
*Ret_sd*	Standard deviation of *Ret* over the fiscal year.
*Roa_sd*	Standard deviation of *Roa* calculated over the last 5 years, with at least 3 years of data required.
*Droa*	Net income in period *t*- net income in period (*t*-1)/total assets in period *t*-1.
*Froa*	Net incomes in period *t* + 1 on assets in period t.
*Occupy*	Other receivables on assets.
*Big4*	The dummy variable of been audited by the Big Four accounting firms.
*Mshare*	Executives' shareholdings on total equities.
*Length*	The logarithm of MD & A's words.

### Statistical description

Table 3 shows the statistical descriptions of the main variables. The average executive tone of our listed companies is 73.2%, revealing that most of the management analysis and discussion of publicly traded corporations use more positive words. From the investor sentiment, the mean is 7.6%, and the standard deviation is 41.8%, revealing there are heterogeneous differences in the investor sentiment over the period.

**Table 3:** Statistical description of the main variables.

**Variable**	**Mean**	**Std.Dev**	**Minimum**	**Maximum**
**Dependent variables**				
*Tone*	0.732	0.121	0.333	0.967
*Manitone*	0.010	0.117	−0.365	0.262
*Realtone*	0.722	0.041	0.582	0.798
**Independent variables**				
*Sentiment*	0.076	0.418	−0.756	0.790
**Moderating variables**				
*Pricepressure*	0.028	0.011	0.010	0.058
*Volumepressure*	0.012	0.012	0.001	0.060
*Expectation*	0.058	0.068	−0.096	0.247
*Gamble*	0.514	0.097	0.282	0.717
**Mediating variables**				
*Overpositive*	0.358	0.480	0.000	1.000
**Control variables**				
*Roa*	0.034	0.061	−0.227	0.210
*Lev*	0.472	0.198	0.071	0.888
*Size*	22.254	1.256	19.768	26.086
*Growth*	0.178	0.462	−0.573	3.134
*Cashflow*	0.047	0.071	−0.164	0.249
*Age*	2.440	0.480	1.386	3.258
*Loss*	0.026	0.158	0.000	1.000
*Btm*	0.636	0.252	0.052	1.246
*Ret*	0.228	0.748	−0.833	6.465
*Ret_sd*	0.131	0.061	0.031	0.638
*Roa_sd*	0.035	0.056	0.002	1.194
*Droa*	0.006	0.073	−0.441	1.148
*Froa*	0.038	0.074	−0.435	0.641
*Occupy*	0.020	0.031	0.000	0.190
*Big4*	0.061	0.240	0.000	1.000
*Mshare*	0.069	0.141	0.000	0.590
*Length*	7.583	0.829	3.912	9.279

## Empirical research

### Empirical models

Given that annual reports of listed companies are generally disclosed in the next year and lag behind capital market investors' expectations and behaviors, we include contemporaneous dependent and independent variables in the empirical models. In addition, market-level investor sentiment (*Sentiment*) does not vary with the firms and has high covariance with annual dummy variables, we thus estimate Equation (7) to link the executive tone and investor sentiment, firm-specific variables, industry, and province dummies to test H1. It is important to note that we include industry and province dummies to control the impacts of industry and district features.

(7) $$\begin{array}{l} Tone_{i,t}\;=\;\alpha_{0}\;+\;\alpha_{1}Sentiment_{t}\;+\;\alpha_{2}Roa_{i,t}\;+\;\alpha_{3}Lev_{i,t}\;+\\ \alpha_{4}Size_{i,t}\;+\;\alpha_{5}Growth_{i,t}\;+\;\alpha_{6}Cashflow_{i,t}\;+\;\alpha_{7}Age_{i,t}\;+\\ \alpha_{8}Loss_{i,t}\;+\;\alpha_{9}Btm_{i,t}+\alpha_{10}Ret_{i,t}\;+\;\alpha_{11}{Ret\text{\_}sd}_{i,t}\;+\\ \alpha_{12}{Roa\text{\_}sd}_{i,t}\;+\;\alpha_{13}Droa_{i,t}\;+\;\alpha_{14}Froa_{i,t}\;+\;\alpha_{15}Occupy_{i,t}\;+\\ \alpha_{16}{Big4}_{i,t}\;+\;\alpha_{17}Mshare_{i,t}\;+\;\alpha_{18}Length_{i,t}\;+\;\sum Industry\\ +\sum Province+\varepsilon_{i,t} \end{array}$$

We estimate Equation (8) to test H2a, H2b, H3a, and H3b.

(8) $$\begin{array}{l} Tone_{i,t}\;=\;\beta_{0}\;+\;\beta_{1}Sentiment_{t}\;+\;\beta_{2}{Mon\text{\_}variables}_{i,t}\;+\\ \beta_{3}Sentiment_{t}*{Mon\text{\_}variables}_{i,t}+\beta_{4}Roa_{i,t}+\beta_{5}Lev_{i,t}\;+\\ \beta_{6}Size_{i,t}\;+\;\beta_{7}Growth_{i,t}\;+\;\beta_{8}Cashflow_{i,t}\;+\;\beta_{9}Age_{i,t}\;+\\ \beta_{10}Loss_{i,t}\;+\;\beta_{11}Btm_{i,t}+\beta_{12}Ret_{i,t}+\beta_{13}{Ret\text{\_}sd}_{i,t}\;+\\ \beta_{14}{Roa\text{\_}sd}_{i,t}\;+\;\beta_{15}Droa_{i,t}\;+\;\beta_{16}Froa_{i,t}\;+\;\beta_{17}Occupy_{i,t}\;+\\ \beta_{18}{Big4}_{i,t}\;+\;\beta_{19}Mshare_{i,t}\;+\;\beta_{20}Length_{i,t}\;+\;\sum Industry\\ +\;\sum Province\;+\;\varepsilon_{i,t} \end{array}$$

where, *Mon*_*variables*_*i,t*_ are the external monitoring variables, including *Pricepressure, Volumepressure, Expectation*, and *Gamble*.

### Empirical results

Table 4 reports the results of benchmark regressions. As shown in Column (1) of Table 4, the coefficient of *Sentiment* is positive at the 1% level, suggesting that executives tend to adopt a more positive intonation to cater to the changes in investor sentiment. This result is strongly consistent with the Catering Theory and supports H1.

**Table 4:** Benchmark regression.

	**(1)**	**(2)**	**(3)**	**(4)**	**(5)**
	***Tone***	***Tone***	***Tone***	***Tone***	***Tone***
*Sentiment*	0.011^***^	0.015^***^	0.011^***^	0.011^***^	0.011^***^
	(5.110)	(6.924)	(5.286)	(4.916)	(5.409)
*Pricepressure*		−0.821^***^			
		(−9.312)			
Sentiment*Pricepressure		0.913^***^			
		(4.633)			
*Volumepressure*			0.012		
			(0.124)		
Sentiment*Volumepressure			0.895^***^		
			(4.927)		
*Expectation*				0.008	
				(0.519)	
Sentiment*Expectation				0.078^***^	
				(2.684)	
*Gamble*					−0.046^***^
					(−3.348)
Sentiment*Gamble					0.041^*^
					(1.871)
*Roa*	0.321^***^	0.320^***^	0.319^***^	0.321^***^	0.293^***^
	(11.324)	(11.354)	(11.263)	(11.350)	(9.997)
*Lev*	0.020^**^	0.022^**^	0.020^**^	0.020^**^	0.021^**^
	(2.150)	(2.397)	(2.134)	(2.170)	(2.313)
*Size*	0.013^***^	0.011^***^	0.013^***^	0.013^***^	0.012^***^
	(8.200)	(6.674)	(8.247)	(8.166)	(7.552)
*Growth*	0.014^***^	0.014^***^	0.014^***^	0.014^***^	0.013^***^
	(6.852)	(6.711)	(6.887)	(6.805)	(6.610)
*Cashflow*	−0.085^***^	−0.084^***^	−0.084^***^	−0.085^***^	−0.086^***^
	(−5.470)	(−5.451)	(−5.427)	(−5.497)	(−5.542)
*Age*	−0.010^***^	−0.011^***^	−0.010^***^	−0.010^***^	−0.009^***^
	(−3.142)	(−3.406)	(−3.085)	(−3.084)	(−2.655)
*Loss*	−0.034^***^	−0.032^***^	−0.034^***^	−0.034^***^	−0.034^***^
	(−4.634)	(−4.427)	(−4.634)	(−4.634)	(−4.691)
*Btm*	−0.057^***^	−0.048^***^	−0.058^***^	−0.057^***^	−0.054^***^
	(−8.839)	(−7.209)	(−8.999)	(−8.740)	(−8.309)
*Ret*	0.004^***^	0.005^***^	0.003^**^	0.004^***^	0.004^***^
	(3.098)	(3.757)	(2.314)	(3.412)	(3.297)
*Ret_sd*	−0.069^***^	−0.036^**^	−0.083^***^	−0.060^***^	−0.062^***^
	(−4.406)	(−2.307)	(−4.951)	(−3.735)	(−3.998)
*Roa_sd*	−0.123^***^	−0.119^***^	−0.123^***^	−0.123^***^	−0.120^***^
	(−4.320)	(−4.238)	(−4.294)	(−4.298)	(−4.239)
*Droa*	−0.037^**^	−0.041^***^	−0.036^**^	−0.038^**^	−0.026
	(−2.431)	(−2.660)	(−2.327)	(−2.452)	(−1.641)
*Froa*	0.058^***^	0.067^***^	0.061^***^	0.057^***^	0.053^***^
	(4.099)	(4.748)	(4.323)	(4.021)	(3.747)
*Occupy*	−0.026	−0.014	−0.026	−0.029	−0.023
	(−0.657)	(−0.352)	(−0.656)	(−0.719)	(−0.574)
*Big4*	−0.005	−0.005	−0.005	−0.005	−0.006
	(−0.903)	(−0.908)	(−0.921)	(−0.878)	(−0.959)
*Mshare*	0.008	0.008	0.009	0.008	0.009
	(0.878)	(0.818)	(0.912)	(0.827)	(0.968)
*Length*	−0.011^***^	−0.010^***^	−0.011^***^	−0.010^***^	−0.010^***^
	(−5.982)	(−5.344)	(−5.879)	(−5.756)	(−5.731)
*_cons*	0.563^***^	0.614^***^	0.561^***^	0.557^***^	0.597^***^
	(16.977)	(17.986)	(16.808)	(16.776)	(17.416)
Industry and province	Yes	Yes	Yes	Yes	Yes
*N*	20,328	20,328	20,328	20,328	20,328
R-squared	0.118	0.122	0.119	0.118	0.119

This table reports the results of benchmark regression. In parentheses, there are the *t*-values of the company clustered robust standard errors. In addition,

*, **, and *** , respectively, represent statistical significance at 10, 5, and 1% levels.

As shown in Column (2) of Table 4, the coefficient of Sentiment^*^Pricepressure is positive at the 1% level, indicating that faced with higher stock price pressure, managers tend to employ a more positive tone to respond to investor sentiment in order to avoid the decline in stock price. This result verifies the Catering Theory from an executive motivation perspective and supports H2a.

As shown in Column (3) of Table 4, the coefficient of Sentiment^*^ *Volumepressure* is positive at the 1% level, revealing that faced with higher trading volume pressure, executives prefer to adopt a more positive tone to cater to investor sentiment to avoid the decline in trading volume. From the perspective of executive motivation, this result validates the Catering Theory and supports H2b.

As shown in Column (4) of Table 4, the coefficient of Sentiment^*^ *Expectation* is positive at the 1% level, indicating that when investor expectation of the corporate is more optimistic, executives tend to use a more positive intonation to respond to investor sentiment and expectation. This result verifies the Catering Theory from an investor expectation perspective and provides strong support for H3a.

As shown in Column (5) of Table 4, the coefficient of Sentiment^*^ *Gamble* is positive at the 10% level, implying that when investor gambling tendency on the stock is higher, managers prefer to adopt a more positive intonation in response to investor sentiment and preference. This result validates the Catering Theory from an investor preference perspective and provides moderate support for H3b.

### Robustness checks

#### 2SLS regressions

We adopt 2SLS regressions to alleviate the quid endogeneity between executive intonation and investor sentiment. Given that economic policy uncertainty can influence investor sentiment and it's difficult for an executive intonation to affect changes and uncertainty in economic policy, we select the variable of China's economic policy uncertainty as the instrumental variable. And then, we calculate the 12-month arithmetic average of the index over the fiscal year using Shangqin Lu and Yun Huang's monthly index of economic policy uncertainty and divide it by 100 to represent the indicator of China's economic policy uncertainty (*Epu*).

Table 5 reports 2SLS regressions. In Column (1), the coefficient of *Epu* is negative at the 1% significant level, indicating that economic policy uncertainty significantly reduces irrational investor sentiment. The coefficient of *Sentiment* is positive at the 10% significant level in Column (2), which is consistent with the previous results. In addition, the Kleibergen-Paap RK LM and Kleibergen-Paap RK Wald F statistics confirm the reasonableness of the instrumental variables.

**Table 5:** 2SLS regressions.

	**(1)**	**(2)**
	***Sentiment***	***Tone***
*Epu*	−0.301^***^	
	(−27.342)	
*Sentiment*		0.033^*^
		(1.740)
*Roa*	−0.665^***^	0.335^***^
	(−9.325)	(10.944)
*Lev*	−0.240^***^	0.025^**^
	(−12.256)	(2.436)
*Size*	0.125^***^	0.010^***^
	(35.306)	(4.056)
*Growth*	0.044^***^	0.013^***^
	(6.689)	(5.854)
*Cashflow*	−0.170^***^	−0.082^***^
	(−3.911)	(−5.184)
*Age*	0.110^***^	−0.012^***^
	(15.391)	(−3.247)
*Loss*	−0.051^***^	−0.033^***^
	(−2.680)	(−4.511)
*Btm*	−0.660^***^	−0.045^***^
	(−42.269)	(−3.825)
*Ret*	0.007	0.003^*^
	(1.572)	(1.645)
*Ret_sd*	0.495^***^	−0.073^***^
	(11.027)	(−4.526)
*Roa_sd*	−0.137^**^	−0.120^***^
	(−2.091)	(−4.286)
*Droa*	0.267^***^	−0.042^***^
	(6.411)	(−2.643)
*Froa*	−0.547^***^	0.071^***^
	(−12.905)	(4.023)
*Occupy*	−0.846^***^	−0.014
	(−8.234)	(−0.342)
*Big4*	−0.075^***^	−0.004
	(−5.564)	(−0.625)
*Mshare*	0.372^***^	0.001
	(15.142)	(0.061)
*Length*	0.020^***^	−0.011^***^
	(5.346)	(−6.006)
*_cons*	−2.270^***^	0.613^***^
	(−35.372)	(11.294)
Industry and province	Yes	Yes
Kleibergen-Paap rk LM statistic		606.58
		0.000
Kleibergen-Paap rk Wald F statistic		747.6
		10%
*N*	20,328	20,328
R-squared	0.180	0.113

This table shows the results of 2SLS regressions. The regressions include other factors and industry dummies. In parentheses, there are the *t*-values of the company clustered robust standard errors. In addition,

*, **, and *** , respectively, represent statistical significance at 10, 5, and 1% levels.

#### Robustness checks for firm fixed-effect models

We adopt firm fixed-effect models to replace original models to alleviate missing variable problems. Table 6 reports the results of the firm fixed-effect models. The regression results provide significant support for the previous results.

**Table 6:** Firm fixed-effect models.

	**(1)**	**(2)**	**(3)**	**(4)**	**(5)**
	***Tone***	***Tone***	***Tone***	***Tone***	***Tone***
*Sentiment*	0.019^***^	0.024^***^	0.019^***^	0.019^***^	0.019^***^
	(8.356)	(10.416)	(8.461)	(8.468)	(8.338)
*Pricepressure*		−0.764^***^			
		(−9.368)			
Sentiment*Pricepressure		0.819^***^			
		(4.302)			
*Volumepressure*			−0.027		
			(−0.335)		
Sentiment*Volumepressure			0.611^***^		
			(3.688)		
*Expectation*				0.050^***^	
				(3.535)	
Sentiment*Expectation				0.081^***^	
				(2.985)	
*Gamble*					0.026^*^
					(1.899)
Sentiment*Gamble					0.033
					(1.533)
*Roa*	0.287^***^	0.288^***^	0.287^***^	0.284^***^	0.297^***^
	(9.859)	(9.971)	(9.865)	(9.775)	(9.926)
*Lev*	0.027^**^	0.030^**^	0.027^**^	0.027^**^	0.026^**^
	(2.256)	(2.574)	(2.267)	(2.308)	(2.180)
*Size*	0.020^***^	0.016^***^	0.020^***^	0.019^***^	0.020^***^
	(6.707)	(5.414)	(6.658)	(6.486)	(6.900)
*Growth*	0.016^***^	0.016^***^	0.016^***^	0.016^***^	0.016^***^
	(8.508)	(8.303)	(8.516)	(8.397)	(8.529)
*Cashflow*	−0.033^**^	−0.031^**^	−0.032^**^	−0.032^**^	−0.033^**^
	(−2.259)	(−2.146)	(−2.211)	(−2.199)	(−2.283)
*Age*	−0.056^***^	−0.058^***^	−0.055^***^	−0.057^***^	−0.058^***^
	(−9.043)	(−9.359)	(−8.783)	(−9.284)	(−9.244)
*Loss*	−0.022^***^	−0.021^***^	−0.022^***^	−0.022^***^	−0.022^***^
	(−3.076)	(−2.886)	(−3.078)	(−3.040)	(−3.073)
*Btm*	−0.101^***^	−0.087^***^	−0.101^***^	−0.100^***^	−0.103^***^
	(−15.268)	(−12.689)	(−15.271)	(−14.976)	(−15.490)
*Ret*	−0.004^***^	−0.003^**^	−0.004^***^	−0.004^***^	−0.004^***^
	(−3.058)	(−2.132)	(−3.365)	(−2.775)	(−3.167)
*Ret_sd*	−0.096^***^	−0.067^***^	−0.104^***^	−0.080^***^	−0.100^***^
	(−6.641)	(−4.651)	(−6.891)	(−5.343)	(−6.912)
*Roa_sd*	−0.053^**^	−0.052^**^	−0.053^**^	−0.050^**^	−0.054^**^
	(−2.449)	(−2.441)	(−2.435)	(−2.333)	(−2.503)
*Droa*	−0.028^*^	−0.031^**^	−0.027^*^	−0.028^*^	−0.032^**^
	(−1.764)	(−1.988)	(−1.713)	(−1.791)	(−1.981)
*Froa*	0.012	0.020	0.015	0.009	0.013
	(0.805)	(1.384)	(0.998)	(0.588)	(0.890)
*Occupy*	−0.103^**^	−0.095^**^	−0.103^**^	−0.102^**^	−0.101^**^
	(−2.237)	(−2.064)	(−2.254)	(−2.226)	(−2.205)
*Big4*	−0.006	−0.006	−0.006	−0.006	−0.005
	(−0.683)	(−0.678)	(−0.668)	(−0.687)	(−0.638)
*Mshare*	0.006	0.006	0.008	0.006	0.007
	(0.304)	(0.290)	(0.392)	(0.273)	(0.325)
*Length*	−0.011^***^	−0.009^***^	−0.011^***^	−0.010^***^	−0.010^***^
	(−4.721)	(−3.804)	(−4.803)	(−4.356)	(−4.646)
*_cons*	0.569^***^	0.640^***^	0.570^***^	0.574^***^	0.546^***^
	(10.591)	(11.626)	(10.593)	(10.713)	(10.006)
*Firm*	Yes	Yes	Yes	Yes	Yes
*N*	20,328	20,328	20,328	20,328	20,328
R-squared	0.098	0.104	0.099	0.099	0.099

This table shows the results of company fixed-effect models. In parentheses, there are the *t*-values of the company clustered robust standard errors. In addition,

*, **, and *** , respectively, represent statistical significance at 10, 5, and 1% levels.

#### Alternative tests of dependent variables

First, there are differences in the length of reports and the importance of words, we thus construct a tone variable using TFIDF methods. We take the TFIDF algorithms to calculate the word's frequency and adopt Equation (1) to calculate the tone variable (*Tone2*). The TFIDF calculation formula is shown in Equation (9).

(9) $$\begin{array}{l} TFIDF_{x,y}\;=\;tf_{x,y}*\log\left(\frac{N}{df_{x}}\right) \end{array}$$

where, *tf*_*x,y*_ is the frequency of word *x* in document *y*; *df*_*x*_ is the number of documents containing word *x*; *N* is the total number of documents.

Next, we calculate the tone indicator (*Tone3*) based on the dictionary constructed by Loughran and McDonald (2011) as the alternative dependent variable. Table 7 shows the results of alternative dependent variables, revealing that the results of the original regressions are robust.

**Table 7:** Alternative tests of dependent variables.

**(A) Alternative dependent variable:** ***Tone2***					
	**(1)**	**(2)**	**(3)**	**(4)**	**(5)**
	***Tone2***	***Tone2***	***Tone2***	***Tone2***	***Tone2***
*Sentiment*	0.027^***^	0.033^***^	0.028^***^	0.027^***^	0.028^***^
	(7.970)	(9.305)	(8.291)	(7.860)	(8.179)
*Pricepressure*		−0.997^***^			
		(−7.101)			
Sentiment*Pricepressure		1.930^***^			
		(6.001)			
*Volumepressure*			−0.185		
			(−1.255)		
Sentiment*Volumepressure			1.444^***^		
			(4.758)		
*Expectation*				0.069^***^	
				(2.861)	
Sentiment*Expectation				0.081^*^	
				(1.759)	
*Gamble*					−0.050^**^
					(−2.332)
Sentiment*Gamble					0.065^*^
					(1.873)
*Roa*	0.340^***^	0.339^***^	0.336^***^	0.337^***^	0.310^***^
	(7.444)	(7.428)	(7.337)	(7.365)	(6.609)
*Lev*	0.012	0.015	0.012	0.013	0.014
	(0.852)	(1.039)	(0.853)	(0.877)	(0.971)
*Size*	0.019^***^	0.017^***^	0.019^***^	0.019^***^	0.018^***^
	(7.746)	(6.532)	(7.590)	(7.526)	(7.281)
*Growth*	0.012^***^	0.012^***^	0.012^***^	0.012^***^	0.011^***^
	(3.783)	(3.673)	(3.830)	(3.664)	(3.598)
*Cashflow*	−0.086^***^	−0.086^***^	−0.086^***^	−0.086^***^	−0.088^***^
	(−3.556)	(−3.531)	(−3.531)	(−3.535)	(−3.611)
*Age*	−0.000	−0.001	0.000	−0.000	0.001
	(−0.058)	(−0.246)	(0.019)	(−0.001)	(0.270)
*Loss*	−0.051^***^	−0.049^***^	−0.051^***^	−0.051^***^	−0.051^***^
	(−4.515)	(−4.349)	(−4.504)	(−4.497)	(−4.550)
*Btm*	−0.069^***^	−0.059^***^	−0.070^***^	−0.067^***^	−0.066^***^
	(−6.845)	(−5.663)	(−6.905)	(−6.530)	(−6.485)
*Ret*	0.004^*^	0.004^*^	0.003	0.005^**^	0.004^**^
	(1.953)	(1.957)	(1.500)	(2.231)	(2.103)
*Ret_sd*	−0.140^***^	−0.108^***^	−0.150^***^	−0.119^***^	−0.133^***^
	(−5.604)	(−4.268)	(−5.607)	(−4.650)	(−5.327)
*Roa_sd*	−0.184^***^	−0.179^***^	−0.182^***^	−0.181^***^	−0.181^***^
	(−4.385)	(−4.290)	(−4.324)	(−4.323)	(−4.330)
*Droa*	−0.005	−0.008	−0.002	−0.006	0.007
	(−0.219)	(−0.326)	(−0.072)	(−0.231)	(0.268)
*Froa*	0.035	0.050^**^	0.039^*^	0.032	0.030
	(1.496)	(2.122)	(1.661)	(1.372)	(1.260)
*Occupy*	−0.078	−0.064	−0.078	−0.076	−0.074
	(−1.243)	(−1.001)	(−1.236)	(−1.195)	(−1.171)
*Big4*	−0.006	−0.006	−0.006	−0.006	−0.006
	(−0.687)	(−0.719)	(−0.696)	(−0.644)	(−0.721)
*Mshare*	0.037^**^	0.037^**^	0.039^**^	0.035^**^	0.038^**^
	(2.340)	(2.339)	(2.456)	(2.207)	(2.401)
*Length*	−0.002	−0.001	−0.002	−0.002	−0.002
	(−0.839)	(−0.387)	(−0.780)	(−0.857)	(−0.663)
*_cons*	0.235^***^	0.296^***^	0.244^***^	0.234^***^	0.272^***^
	(4.546)	(5.523)	(4.686)	(4.514)	(5.082)
Industry and province	Yes	Yes	Yes	Yes	Yes
*N*	20,328	20,328	20,328	20,328	20,328
R-squared	0.096	0.100	0.097	0.097	0.097
**(B) Alternative dependent variable:** ***Tone3***					
	**(1)**	**(2)**	**(3)**	**(4)**	**(5)**
	***Tone3***	***Tone3***	***Tone3***	***Tone3***	***Tone3***
*Sentiment*	0.016^***^	0.020^***^	0.017^***^	0.015^***^	0.017^***^
	(4.619)	(5.709)	(4.983)	(4.557)	(5.046)
*Pricepressure*		−0.816^***^			
		(−5.904)			
Sentiment*Pricepressure		1.203^***^			
		(4.134)			
*Volumepressure*			−0.224		
			(−1.511)		
Sentiment*Volumepressure			1.318^***^		
			(4.679)		
*Expectation*				0.021	
				(0.832)	
Sentiment*Expectation				0.033	
				(0.747)	
*Gamble*					−0.109^***^
					(−4.773)
Sentiment*Gamble					0.072^**^
					(2.189)
*Roa*	0.781^***^	0.780^***^	0.776^***^	0.780^***^	0.717^***^
	(17.267)	(17.245)	(17.138)	(17.238)	(15.671)
*Lev*	0.064^***^	0.066^***^	0.064^***^	0.064^***^	0.067^***^
	(4.066)	(4.203)	(4.076)	(4.073)	(4.282)
*Size*	0.015^***^	0.013^***^	0.015^***^	0.015^***^	0.013^***^
	(5.335)	(4.509)	(5.119)	(5.277)	(4.555)
*Growth*	0.047^***^	0.047^***^	0.048^***^	0.047^***^	0.046^***^
	(13.546)	(13.436)	(13.564)	(13.516)	(13.271)
*Cashflow*	−0.177^***^	−0.177^***^	−0.177^***^	−0.177^***^	−0.179^***^
	(−7.105)	(−7.086)	(−7.088)	(−7.098)	(−7.234)
*Age*	−0.036^***^	−0.037^***^	−0.035^***^	−0.036^***^	−0.032^***^
	(−6.730)	(−6.870)	(−6.662)	(−6.718)	(−6.024)
*Loss*	−0.064^***^	−0.062^***^	−0.064^***^	−0.064^***^	−0.065^***^
	(−5.933)	(−5.776)	(−5.900)	(−5.926)	(−6.051)
*Btm*	−0.097^***^	−0.088^***^	−0.097^***^	−0.096^***^	−0.089^***^
	(−9.441)	(−8.249)	(−9.482)	(−9.225)	(−8.694)
*Ret*	0.016^***^	0.016^***^	0.015^***^	0.016^***^	0.016^***^
	(7.744)	(7.701)	(7.320)	(7.857)	(8.044)
*Ret_sd*	−0.234^***^	−0.205^***^	−0.241^***^	−0.227^***^	−0.219^***^
	(−9.966)	(−8.653)	(−9.515)	(−9.224)	(−9.280)
*Roa_sd*	−0.289^***^	−0.284^***^	−0.287^***^	−0.288^***^	−0.281^***^
	(−6.029)	(−5.986)	(−5.957)	(−6.010)	(−5.923)
*Droa*	−0.097^***^	−0.100^***^	−0.094^***^	−0.098^***^	−0.071^***^
	(−4.099)	(−4.215)	(−3.923)	(−4.106)	(−2.952)
*Froa*	0.164^***^	0.174^***^	0.167^***^	0.163^***^	0.152^***^
	(7.630)	(8.125)	(7.798)	(7.577)	(7.093)
*Occupy*	0.023	0.035	0.024	0.024	0.030
	(0.367)	(0.559)	(0.379)	(0.376)	(0.478)
*Big4*	−0.032^***^	−0.032^***^	−0.032^***^	−0.032^***^	−0.033^***^
	(−2.823)	(−2.832)	(−2.829)	(−2.808)	(−2.893)
*Mshare*	0.041^**^	0.040^**^	0.043^***^	0.040^**^	0.043^***^
	(2.470)	(2.448)	(2.597)	(2.425)	(2.599)
*Length*	−0.012^***^	−0.011^***^	−0.012^***^	−0.012^***^	−0.011^***^
	(−4.049)	(−3.666)	(−4.012)	(−4.002)	(−3.735)
*_cons*	0.260^***^	0.310^***^	0.271^***^	0.259^***^	0.339^***^
	(4.429)	(5.164)	(4.549)	(4.398)	(5.588)
Industry and province	Yes	Yes	Yes	Yes	Yes
*N*	20,328	20,328	20,328	20,328	20,328
R-squared	0.213	0.215	0.214	0.213	0.216

Panel A and Panel B, respectively, show the results of alternative dependent variables Tone2 and Tone3. The regressions include other factors and industry dummies. In parentheses, there are the *t*-values of the company clustered robust standard errors. In addition,

*, **, and *** , respectively, represent statistical significance at 10, 5, and 1% levels.

#### Alternative tests of independent variables

The Chinese capital market primarily adopts two indicators, the China investor composite sentiment index (CICSI) and Investor Sentiment Index (ISI), as indicators for measuring investor sentiment. We collect the monthly index of CICSI from the CSMAR database. Then, we calculate the annual arithmetic average of the index to measure the investor sentiment (*CICSI*) and substitute the original independent variables. The findings, which are presented in Table 8, significantly attest to the main regressions' robustness.

**Table 8:** Alternative tests of independent variables.

	**(1)**	**(2)**	**(3)**	**(4)**	**(5)**
	***Tone***	***Tone***	***Tone***	***Tone***	***Tone***
*CISSI*	0.010^***^	0.010^***^	0.009^***^	0.010^***^	0.010^***^
	(7.194)	(7.271)	(6.743)	(7.593)	(7.359)
*Pricepressure*		−0.958^***^			
		(−11.240)			
CISSI*Pricepressure		0.436^***^			
		(3.928)			
*Volumepressure*			−0.092		
			(−0.972)		
CISSI*Volumepressure			0.520^***^		
			(5.305)		
*Expectation*				0.028^*^	
				(1.841)	
CISSI*Expectation				0.028	
				(1.527)	
*Gamble*					−0.059^***^
					(−4.292)
CISSI*Gamble					0.025^*^
					(1.958)
*Roa*	0.376^***^	0.365^***^	0.375^***^	0.374^***^	0.338^***^
	(13.801)	(13.430)	(13.757)	(13.691)	(11.853)
*Lev*	0.014	0.017^*^	0.015	0.015	0.016^*^
	(1.549)	(1.884)	(1.583)	(1.595)	(1.785)
*Size*	0.006^***^	0.005^***^	0.006^***^	0.006^***^	0.006^***^
	(4.171)	(3.292)	(3.991)	(4.100)	(3.695)
*Growth*	0.015^***^	0.014^***^	0.015^***^	0.014^***^	0.014^***^
	(7.152)	(7.050)	(7.185)	(7.053)	(6.874)
*Cashflow*	−0.085^***^	−0.084^***^	−0.084^***^	−0.085^***^	−0.086^***^
	(−5.491)	(−5.451)	(−5.432)	(−5.484)	(−5.567)
*Age*	0.004	0.004	0.004	0.004	0.005
	(1.003)	(0.909)	(1.011)	(1.130)	(1.350)
*Loss*	−0.028^***^	−0.027^***^	−0.027^***^	−0.028^***^	−0.028^***^
	(−3.797)	(−3.691)	(−3.760)	(−3.800)	(−3.926)
*Btm*	−0.033^***^	−0.035^***^	−0.033^***^	−0.034^***^	−0.032^***^
	(−5.794)	(−6.085)	(−5.770)	(−5.872)	(−5.632)
*Ret*	0.007^***^	0.009^***^	0.007^***^	0.007^***^	0.007^***^
	(5.864)	(6.897)	(5.704)	(5.934)	(5.913)
*Ret_sd*	−0.058^***^	−0.026^*^	−0.071^***^	−0.049^***^	−0.053^***^
	(−3.771)	(−1.715)	(−4.243)	(−3.090)	(−3.400)
*Roa_sd*	−0.108^***^	−0.107^***^	−0.107^***^	−0.107^***^	−0.105^***^
	(−4.032)	(−4.004)	(−3.975)	(−3.988)	(−3.941)
*Droa*	−0.055^***^	−0.055^***^	−0.054^***^	−0.055^***^	−0.040^**^
	(−3.687)	(−3.694)	(−3.590)	(−3.669)	(−2.565)
*Froa*	0.075^***^	0.079^***^	0.076^***^	0.073^***^	0.067^***^
	(5.325)	(5.628)	(5.448)	(5.202)	(4.754)
*Occupy*	−0.025	−0.009	−0.023	−0.025	−0.018
	(−0.619)	(−0.224)	(−0.565)	(−0.628)	(−0.450)
*Big4*	−0.003	−0.004	−0.003	−0.003	−0.004
	(−0.570)	(−0.636)	(−0.554)	(−0.537)	(−0.667)
*Mshare*	0.011	0.011	0.011	0.010	0.012
	(1.160)	(1.132)	(1.103)	(1.064)	(1.289)
*Length*	−0.006^***^	−0.005^***^	−0.006^***^	−0.006^***^	−0.006^***^
	(−3.209)	(−2.672)	(−3.031)	(−3.073)	(−3.099)
*_cons*	0.692^***^	0.740^***^	0.697^***^	0.690^***^	0.729^***^
	(19.764)	(20.612)	(19.692)	(19.636)	(20.354)
Industry and province	Yes	Yes	Yes	Yes	Yes
*N*	20,328	20,328	20,328	20,328	20,328
R-squared	0.114	0.120	0.115	0.114	0.116

This table reports regression results of the alternative independent variables. The regressions include other factors and industry dummies. In parentheses, there are the *t*-values of the company clustered robust standard errors. In addition,

*, **, and *** , respectively, represent statistical significance at 10, 5, and 1% levels.

#### Robustness checks for censored samples

When a company is going through a bankruptcy proceeding, its business decisions will differ from normal enterprises. We exclude the samples in the bankruptcy process for robustness checks. We follow the approach of Altman (2013) to calculate the bankruptcy risk indicator (*Zscore*) and exclude the samples whose *Zscore* values are lower than 1.81.

(10) $$\begin{array}{l} Zscore_{i,t}\;=\;1.2{O\text{\_}capital}_{i,t}\;+\;1.4{R\text{\_}Earning}_{i,t}+3.3Equity_{i,t}+\\ 0.6Ebit_{i,t}+1.0Ros_{i,t} \end{array}$$

where, *O*_*capital*_*i,t*_ is the working capital on total assets; *R*_*earning*_*i,t*_ is retained earnings on total assets; *Equity*_*i,t*_ is shareholders' equities on total assets; *Ebit*_*i,t*_ is the earnings before interest and tax; *Ros*_*i,t*_ is the operating income on total assets.

Considering that the financial crisis may have a heterogeneous effect on executive tone, we thus delete the firm-year samples of 2008 and 2014 for regressions. Table 9 reveals the regression results of censored samples, which strongly verify the robustness of previous results.

**Table 9:** Robustness checks for censored samples.

**(A) Delete bankruptcy risk firm-year samples**					
	**(1)**	**(2)**	**(3)**	**(4)**	**(5)**
	***Tone***	***Tone***	***Tone***	***Tone***	***Tone***
*Sentiment*	0.009^***^	0.013^***^	0.010^***^	0.009^***^	0.010^***^
	(4.157)	(5.820)	(4.321)	(3.970)	(4.409)
*Pricepressure*		−0.804^***^			
		(−8.626)			
Sentiment*Pricepressure		0.871^***^			
		(4.260)			
*Volumepressure*			0.019		
			(0.191)		
Sentiment*Volumepressure			0.787^***^		
			(4.108)		
*Expectation*				0.005	
				(0.334)	
Sentiment*Expectation				0.105^***^	
				(3.419)	
*Gamble*					−0.040^***^
					(−2.744)
Sentiment*Gamble					0.019
					(0.872)
*Roa*	0.305^***^	0.306^***^	0.303^***^	0.306^***^	0.280^***^
	(9.925)	(9.981)	(9.863)	(9.969)	(8.822)
*Lev*	0.024^**^	0.026^***^	0.023^**^	0.024^**^	0.024^***^
	(2.506)	(2.722)	(2.480)	(2.537)	(2.600)
*Size*	0.013^***^	0.011^***^	0.013^***^	0.013^***^	0.012^***^
	(7.623)	(6.254)	(7.663)	(7.615)	(7.092)
*Growth*	0.016^***^	0.015^***^	0.016^***^	0.016^***^	0.015^***^
	(6.959)	(6.800)	(6.989)	(6.906)	(6.754)
*Cashflow*	−0.079^***^	−0.078^***^	−0.079^***^	−0.080^***^	−0.080^***^
	(−4.871)	(−4.817)	(−4.852)	(−4.903)	(−4.908)
*Age*	−0.009^***^	−0.010^***^	−0.009^***^	−0.009^***^	−0.008^**^
	(−2.672)	(−2.915)	(−2.621)	(−2.598)	(−2.277)
*Loss*	−0.025^***^	−0.024^***^	−0.025^***^	−0.025^***^	−0.026^***^
	(−2.995)	(−2.873)	(−3.006)	(−2.992)	(−3.076)
*Btm*	−0.055^***^	−0.046^***^	−0.055^***^	−0.055^***^	−0.052^***^
	(−8.191)	(−6.620)	(−8.323)	(−8.151)	(−7.737)
*Ret*	0.003^**^	0.005^***^	0.003^*^	0.004^***^	0.004^***^
	(2.537)	(3.216)	(1.937)	(2.913)	(2.706)
*Ret_sd*	−0.061^***^	−0.030^*^	−0.074^***^	−0.051^***^	−0.056^***^
	(−3.782)	(−1.806)	(−4.246)	(−3.063)	(−3.446)
*Roa_sd*	−0.162^***^	−0.158^***^	−0.162^***^	−0.161^***^	−0.159^***^
	(−4.372)	(−4.316)	(−4.357)	(−4.346)	(−4.312)
*Droa*	−0.044^***^	−0.047^***^	−0.042^**^	−0.044^***^	−0.034^*^
	(−2.590)	(−2.792)	(−2.501)	(−2.624)	(−1.926)
*Froa*	0.046^***^	0.055^***^	0.049^***^	0.045^***^	0.042^***^
	(3.060)	(3.651)	(3.251)	(2.968)	(2.782)
*Occupy*	0.024	0.037	0.023	0.021	0.027
	(0.521)	(0.791)	(0.497)	(0.460)	(0.577)
*Big4*	−0.004	−0.004	−0.004	−0.004	−0.005
	(−0.642)	(−0.652)	(−0.669)	(−0.611)	(−0.717)
*Mshare*	0.013	0.012	0.013	0.012	0.013
	(1.320)	(1.271)	(1.343)	(1.280)	(1.392)
*Length*	−0.013^***^	−0.011^***^	−0.012^***^	−0.012^***^	−0.012^***^
	(−6.551)	(−5.951)	(−6.469)	(−6.279)	(−6.334)
*_cons*	0.567^***^	0.618^***^	0.565^***^	0.560^***^	0.597^***^
	(15.943)	(16.912)	(15.735)	(15.702)	(16.182)
Industry and province	Yes	Yes	Yes	Yes	Yes
*N*	18,338	18,338	18,338	18,338	18,338
R-squared	0.107	0.111	0.108	0.108	0.108
**(B) Delete financial crisis firm-year samples**					
	**(1)**	**(2)**	**(3)**	**(4)**	**(5)**
	***Tone***	***Tone***	***Tone***	***Tone***	***Tone***
*Sentiment*	0.012^***^	0.018^***^	0.013^***^	0.011^***^	0.014^***^
	(3.948)	(5.375)	(4.198)	(3.630)	(4.528)
*Pricepressure*		−1.089^***^			
		(−10.428)			
Sentiment*Pricepressure		0.610^*^			
		(1.647)			
*Volumepressure*			−0.053		
			(−0.497)		
Sentiment*Volumepressure			0.404		
			(1.380)		
*Expectation*				0.026	
				(1.474)	
Sentiment*Expectation				0.276^***^	
				(6.527)	
*Gamble*					−0.054^***^
					(−3.574)
Sentiment*Gamble					0.075^**^
					(2.389)
*Roa*	0.311^***^	0.306^***^	0.311^***^	0.306^***^	0.280^***^
	(10.107)	(10.009)	(10.101)	(9.967)	(8.821)
*Lev*	0.022^**^	0.024^**^	0.022^**^	0.022^**^	0.024^**^
	(2.189)	(2.427)	(2.218)	(2.221)	(2.394)
*Size*	0.013^***^	0.010^***^	0.013^***^	0.014^***^	0.012^***^
	(7.987)	(6.017)	(7.796)	(8.111)	(7.242)
*Growth*	0.015^***^	0.015^***^	0.015^***^	0.015^***^	0.014^***^
	(6.818)	(6.728)	(6.847)	(6.588)	(6.517)
*Cashflow*	−0.082^***^	−0.080^***^	−0.082^***^	−0.083^***^	−0.083^***^
	(−4.593)	(−4.511)	(−4.591)	(−4.667)	(−4.663)
*Age*	−0.013^***^	−0.013^***^	−0.013^***^	−0.011^***^	−0.011^***^
	(−3.468)	(−3.648)	(−3.488)	(−3.115)	(−3.031)
*Loss*	−0.026^***^	−0.025^***^	−0.026^***^	−0.025^***^	−0.027^***^
	(−4.063)	(−3.871)	(−4.050)	(−3.950)	(−4.184)
*Btm*	−0.064^***^	−0.051^***^	−0.063^***^	−0.067^***^	−0.060^***^
	(−8.845)	(−6.693)	(−8.692)	(−9.083)	(−8.292)
*Ret*	−0.005^***^	−0.001	−0.005^**^	−0.005^***^	−0.005^***^
	(−2.768)	(−0.628)	(−2.497)	(−2.879)	(−2.737)
*Ret_sd*	−0.040^**^	−0.004	−0.041^**^	−0.014	−0.029
	(−2.119)	(−0.221)	(−2.041)	(−0.696)	(−1.546)
*Roa_sd*	−0.123^***^	−0.115^***^	−0.122^***^	−0.120^***^	−0.119^***^
	(−3.970)	(−3.861)	(−3.955)	(−3.890)	(−3.889)
*Droa*	−0.039^**^	−0.044^***^	−0.039^**^	−0.037^**^	−0.027
	(−2.415)	(−2.673)	(−2.400)	(−2.290)	(−1.578)
*Froa*	0.063^***^	0.070^***^	0.063^***^	0.061^***^	0.058^***^
	(3.951)	(4.367)	(3.943)	(3.807)	(3.625)
*Occupy*	−0.064	−0.042	−0.062	−0.076^*^	−0.056
	(−1.443)	(−0.948)	(−1.403)	(−1.700)	(−1.250)
*Big4*	−0.007	−0.006	−0.007	−0.007	−0.007
	(−1.130)	(−1.051)	(−1.105)	(−1.130)	(−1.195)
*Mshare*	0.021^**^	0.020^*^	0.020^**^	0.021^**^	0.022^**^
	(2.016)	(1.939)	(1.962)	(2.052)	(2.102)
*Length*	−0.016^***^	−0.014^***^	−0.016^***^	−0.014^***^	−0.015^***^
	(−8.167)	(−7.057)	(−8.196)	(−7.337)	(−7.952)
*_cons*	0.598^***^	0.665^***^	0.605^***^	0.571^***^	0.640^***^
	(16.476)	(17.781)	(16.445)	(15.542)	(17.061)
Industry and province	Yes	Yes	Yes	Yes	Yes
*N*	15,656	15,656	15,656	15,656	15,656
R-squared	0.119	0.125	0.119	0.121	0.120

This table shows the results of the robustness checks for censored samples. The regressions include other factors and industry dummies. In parentheses, there are the *t*-values of the company clustered robust standard errors. In addition,

*, **, and *** , respectively, represent statistical significance at 10, 5, and 1% levels.

## Further research

### Intermediary mechanism test

From the perspective of the Catering Theory, investor sentiment is the primary determination that rational executives make their financial decisions (Baker and Wurgler, 2004b). However, Hirshleifer and Teoh (2009) argue that irrational investor sentiment can influence executives to generate over-optimistic emotions, which result in more positive behaviors and activities, from the Emotional contagion Theory. As Barger and Grandey (2006) consider, emotional contagion is the process by which an individual or group influences others through emotional states and behavioral attitudes. In this case, we wonder if intonation catering behavior is the validation of the Catering Theory or the Emotional Contagion Theory.

In further research, we perform intermediary regressions to test the mechanism by which investor sentiment influences executive tone (Equations 7, 11, 12). We use the dummy variable of over-valuation of earnings forecast to measure the degree of executive optimism (*Overpositive*). If the actual company profit level is lower than the estimated earnings level at least once in the sample period in 1Q, 6Q, 3Q, and yearly reports, the variable of *OverPositive* takes the value of 1. Otherwise, it takes the value of 0.

(11) $$\begin{array}{l} Overpositive_{i,t}\;=\;\alpha_{0}+\delta_{1}Sentiment_{t}\;+\;\delta_{2}Roa_{i,t}+\delta_{3}Lev_{i,t}\;+\\ \delta_{4}Size_{i,t}\;+\;\delta_{5}Growth_{i,t}\;+\;\delta_{6}Cashflow_{i,t}+\delta_{7}Age_{i,t}\;+\\ \delta_{8}Loss_{i,t}\;+\;\delta_{9}Btm_{i,t}\;+\;\delta_{10}Ret_{i,t}+\delta_{11}{Ret\text{\_}sd}_{i,t}\;+\\ \delta_{12}{Roa\text{\_}sd}_{i,t}\;+\;\delta_{13}Droa_{i,t}+\delta_{14}Froa_{i,t}+\delta_{15}Occupy_{i,t}\;+\\ \delta_{16}{Big4}_{i,t}\;+\;\delta_{17}Mshare_{i,t}\;+\;\delta_{18}Length_{i,t}+\sum Industry\\ +\;\sum Province+\varepsilon_{i,t} \end{array}$$

(12) $$\begin{array}{l} Tone_{i,t}=\gamma_{0}\;+\gamma_{1}Overpositive_{i,t}\;+\;\gamma_{2}Sentiment_{t}\;+\\ \gamma_{3}Roa_{i,t}\;+\;\gamma_{4}Lev_{i,t}\;+\;\gamma_{5}Size_{i,t}\;+\;\gamma_{6}Growth_{i,t}\;+\\ \gamma_{7}Cashflow_{i,t}\;+\;\gamma_{8}Age_{i,t}\;+\;\gamma_{9}Loss_{i,t}\;+\;\gamma_{10}Btm_{i,t}\;+\\ \gamma_{11}Ret_{i,t}+\;\gamma_{12}{Ret\text{\_}sd}_{i,t}\;+\;\gamma_{13}{Roa\text{\_}sd}_{i,t}+\gamma_{14}Droa_{i,t}\;+\\ \gamma_{15}Froa_{i,t}\;+\;\gamma_{16}Occupy_{i,t}\;+\;\gamma_{17}{Big4}_{i,t}\;+\;\gamma_{18}Mshare_{i,t}\;+\\ \gamma_{19}Length_{i,t}\;+\;\sum Industry\;+\\ \sum Province\;+\;\varepsilon_{i,t} \end{array}$$

Table 10 reports the results of intermediary mechanism tests. In Column (2), the coefficient of *Sentiment* is positive at the 1% significant level, demonstrating that irrational investor sentiment infected executives, generating over-optimistic emotions. However, in Column (3) of Table 10, the coefficient of *Overpositive* is positive but not significant, demonstrating that the relationship between investor sentiment (*Sentiment*) and executive tone (*Tone*) is not mediated by executive optimism (*OverPositive*). These results show that intonation catering is a tactic made by rational executives and is consistent with the Catering Theory.

**Table 10:** Intermediary mechanism tests.

	**(1)**	**(2)**	**(3)**
	***Tone***	***Overpositive***	***Tone***
*Sentiment*	0.011^***^	0.570^***^	0.011^***^
	(5.110)	(13.257)	(4.979)
*Overpositive*			0.003
			(1.341)
*Roa*	0.321^***^	10.069^***^	0.316^***^
	(11.324)	(15.713)	(11.086)
*Lev*	0.020^**^	0.643^***^	0.020^**^
	(2.150)	(4.711)	(2.119)
*Size*	0.013^***^	−0.039	0.013^***^
	(8.201)	(−1.515)	(8.201)
*Growth*	0.014^***^	0.587^***^	0.014^***^
	(6.852)	(12.153)	(6.664)
*Cashflow*	−0.085^***^	−0.350	−0.085^***^
	(−5.470)	(−1.204)	(−5.467)
*Age*	−0.010^***^	−0.652^***^	−0.010^***^
	(−3.142)	(−12.802)	(−3.021)
*Loss*	−0.034^***^	−0.608^***^	−0.034^***^
	(−4.634)	(−3.071)	(−4.625)
*Btm*	−0.057^***^	0.127	−0.057^***^
	(−8.840)	(1.159)	(−8.833)
*Ret*	0.004^***^	0.071^***^	0.004^***^
	(3.098)	(2.697)	(3.067)
*Ret_sd*	−0.069^***^	0.089	−0.069^***^
	(−4.406)	(0.284)	(−4.399)
*Roa_sd*	−0.123^***^	−1.049^**^	−0.123^***^
	(−4.320)	(−2.215)	(−4.313)
*Droa*	−0.037^**^	2.163^***^	−0.038^**^
	(−2.430)	(4.496)	(−2.472)
*Froa*	0.058^***^	−0.022	0.058^***^
	(4.099)	(−0.075)	(4.083)
*Occupy*	0.008	1.280^***^	0.008
	(0.879)	(7.382)	(0.794)
*Big4*	−0.026	−0.376	−0.027
	(−0.656)	(−0.520)	(−0.662)
*Mshare*	−0.005	−0.336^***^	−0.005
	(−0.903)	(−3.212)	(−0.874)
*Length*	−0.011^***^	0.234^***^	−0.011^***^
	(−5.983)	(8.586)	(−6.053)
*_cons*	0.563^***^	−1.325^***^	0.562^***^
	(16.977)	(−2.681)	(16.953)
Industry and province	Yes	Yes	Yes
*N*	20,328	20,328	20,328
R-squared	0.118	0.142	0.118

This table shows the results of Intermediary mechanism tests. The regressions include other factors and industry dummies. In parentheses, there are the *t*-values of the company clustered robust standard errors. In addition,

*, **, and *** , respectively, represent statistical significance at 10, 5, and 1% levels.

#### Intonation tactic analysis

From the perspective of the Catering Theory, executives would like to adopt various activities and tactics to cater to investor sentiment (Kong, 2018; Elbannan, 2020). According to Huang et al. (2014), executive intonation can divide into the real (narrative disclosures based on the firm's performance and earnings) and manipulated tones (over-optimistic narrative disclosures manipulated by managers). Rational executives tend to make decisions depending on balancing returns and costs and choose appropriate times and methods to achieve their specific goals. In this case, we believe that executives may adopt different intonation catering tactics to respond to irrational investors.

According to the approach of Huang et al. (2014), we construct a fitted model of the executive tone (Equation 13) for regressions and adopt the residual as the manipulated tone (*Mani_Tone*) and the fitted value as the real tone (*Real_Tone*), We then construct Equation (14) using *Mani_Tone* and *Real_Tone* as dependent variables for empirical regressions.

(13) $$\begin{array}{l} Tone_{i,t}=\theta_{0}\;+\;\theta_{1}Roa_{i,t}\;+\;\theta_{2}Ret_{i,t}\;+\;\theta_{3}Size_{i,t}\;+\\ \theta_{4}Btm_{i,t}\;+\theta_{5}{Ret\text{\_}sd}_{i,t}\;+\;\theta_{6}{Roa\text{\_}sd}_{i,t}\;+\;\theta_{7}Age_{i,t}\;+\\ \theta_{8}Loss_{i,t}+\theta_{9}{D\text{\_}Roa}_{i,t}+\varepsilon_{i,t} \end{array}$$

(14) $$\begin{array}{l} {Tone\text{\_}tactics}_{i,t}=\mu_{0}\;+\;\mu_{1}Sentiment_{t}\;+\;\mu_{2}Roa_{i,t}\;+\\ \mu_{3}Lev_{i,t}+\;\mu_{4}Size_{i,t}\;+\;\mu_{5}Growth_{i,t}\;+\;\mu_{6}Cashflow_{i,t}\;+\\ \mu_{7}Age_{i,t}\;+\mu_{8}Loss_{i,t}\;+\;\mu_{9}Btm_{i,t}\;+\;\mu_{10}Ret_{i,t}\;+\\ \mu_{11}{Ret\text{\_}sd}_{i,t}+\mu_{12}{Roa\text{\_}sd}_{i,t}\;+\;\mu_{13}Droa_{i,t}\;+\;\mu_{14}{Froa}_{i,t}\;+\\ \mu_{15}Occupy_{i,t}\;+\mu_{16}{Big4}_{i,t}\;+\;\mu_{17}Mshare_{i,t}+\\ \mu_{18}Length_{i,t}\;+\sum Industry+\;\sum Province\;+\;\varepsilon_{i,t} \end{array}$$

where, *Tone*_*tactics*_*i, t*_ refer to the intonation tactic variables, including *Mani_Tone* and *Real_Tone*.

Table 11 reveals the results of the intonation tactic analysis. As shown in Columns (1) and (2), the coefficients of *Mani_Tone* and *Real_Tone* are both positive at the 1% level, indicating that executives tend to adopt real and manipulated tone disclosures to cater to investors' emotions. In addition, the coefficients of *Mani_Tone* and *Real_Tone* are 0.11 and 0.001 indicating that investor sentiment rises one unit can result in 0.11 and 0.001 unit increases for *Mani_Tone* and *Real_Tone*. These results suggest that as investor irrational sentiment increases, executives tend to increase manipulated and real tones in different proportions to respond to investor sentiment.

**Table 11:** Intonation tactics analysis.

	**(1)**	**(2)**
	***Manitone***	***Realtone***
*Sentiment*	0.011^***^	0.001^***^
	(5.105)	(3.479)
*Roa*	−0.065^**^	0.377^***^
	(−2.324)	(85.374)
*Lev*	0.019^**^	0.001
	(2.018)	(1.338)
*Size*	−0.005^***^	0.018^***^
	(−3.003)	(96.854)
*Growth*	0.016^***^	−0.001^***^
	(7.630)	(−5.350)
*Cashflow*	−0.071^***^	−0.011^***^
	(−4.598)	(−5.875)
*Age*	0.001	−0.011^***^
	(0.249)	(−37.092)
*Loss*	−0.006	−0.030^***^
	(−0.764)	(−29.257)
*Btm*	0.007	−0.065^***^
	(1.089)	(−84.657)
*Ret*	−0.003^***^	0.008^***^
	(−2.628)	(42.325)
*Ret_sd*	−0.040^**^	−0.028^***^
	(−2.559)	(−12.861)
*Roa_sd*	0.049^**^	−0.152^***^
	(1.976)	(−34.863)
*Droa*	−0.032^**^	−0.009^***^
	(−2.067)	(−3.161)
*Froa*	0.082^***^	−0.021^***^
	(5.668)	(−8.947)
*Occupy*	−0.035	−0.006
	(−0.866)	(−1.411)
*Big4*	−0.005	−0.001
	(−0.780)	(−1.301)
*Mshare*	0.010	−0.002^*^
	(1.094)	(−1.908)
*Length*	−0.011^***^	0.001^***^
	(−5.831)	(3.270)
*_cons*	0.167^***^	0.388^***^
	(5.042)	(114.350)
Industry and province	Yes	Yes
*N*	20,328	20,328
R-squared	0.053	0.879

This table shows the results of intonation tactics analyses, which distinguish the Real Executive Tone and Manipulated Executive Tone. The regressions include other factors and industry dummies. In parentheses, there are the *t*-values of the company clustered robust standard errors. In addition,

*, **, and *** , respectively, represent statistical significance at 10, 5, and 1% levels.

## Discussion

Adopting the Catering Theory perspective, we conduct an empirical analysis to investigate the association between investor sentiment and executive tone; we also conduct moderating regressions to determine whether executives' incentives and investors' behaviors can influence managers catering activities. Finally, in the Section Further research, we attempt to clarify the mechanism by which investor sentiment influences executive tone. We outline the results of these analyses below.

First, our findings verify the Catering Theory from the perspective of executive tone. The empirical results show a positive correlation between market-wide investor sentiment and executive tone. This finding indicates that managers tend to pay attention to market-wide investor sentiments and regard them as the determinants of intonation disclosures; this finding is in line with the findings of previous Catering Theory research (Mian and Sankaraguruswamy, 2012; Simpson, 2013).

Second, we conduct moderating regressions to determine whether executive incentives and firm-level investor behaviors influence the catering decisions of managers. The empirical results show that stock price and trading volume pressures on executives, and firm-level investor expectation and gambling tendency, positively moderate the positive correlation between investor sentiment and executive tone. These findings demonstrate that rational managers tend to change intonation tactics based on comprehensive judgments of subjective and objective factors and internal and external conditions.

Third, we attempt to clarify the mechanism of investor sentiment influencing executive tone. The results show that executive optimism does not mediate the association between investor sentiment and executive tone. This finding indicates that intonation catering is a rational tactic executives deploy and provides strong evidence for the Catering Theory. In addition, we find that executives tend to increase manipulated and real tones in different proportions to cater to changes in investor sentiment. These results demonstrate that intonation can be strategically controlled and even manipulated by managers to respond to investor sentiment.

## Theoretical implications

First, this study attempts to verify the Catering Theory from a comprehensive perspective. Few past studies investigate how executives detect irrational investor sentiment and what types of investor performance and reactions can be used as foundations for making catering decisions. Our research finds that executives may focus on investor market- or firm-related sentiment, expectations, and preferences, which can influence their decision-making regarding catering activities.

Second, few previous studies examine the motivations that incentivize executives to cater to investor sentiment. We find that managers tend to respond to irrational investors by seeking to avoid stock price and trading volume declines—a finding that complements and extends previous research on the motivations related to the Catering Theory.

Third, previous studies primarily focus on the methods and activities adopted by managers to respond to investor sentiment (Chang et al., 2007; Elbannan, 2020). Additionally, other research demonstrates that financial information can be manipulated through earnings management to effectively respond to investors (Mian and Sankaraguruswamy, 2012; Simpson, 2013; Kong, 2018). As a kind of narrative disclosure, we find that executives can disclose strategically and even manipulate intonation to cater to irrational investors—a finding that extends research on information-manipulated methods for the Catering Theory.

Fourth, our findings expand the research on catering behavior mechanisms. While a number of previous studies regard catering behavior as the choice of rational executives (Mian and Sankaraguruswamy, 2012; Elbannan, 2020), other researchers find that it is caused by emotional contagion (Hirshleifer and Teoh, 2009). In this study, we find that intonation is a catering tactic deployed by rational managers to achieve specific goals rather than the result of over-optimistic emotions.

## Practical implications

First, regulators should continuously strengthen the supervision of firms' information disclosures. Our findings reveal that managers can use narrative disclosures to achieve their specific goals. Therefore, regulations requiring that narrative disclosures specify sentence and word usage standards should be improved. In addition, a number of Chinese listed firms promoted Internet finance and blockchain concepts, trapping many investors and leading them to lose large amounts of money. Thus, in situations involving irrational investors and frequent transactions, regulators need to pay more attention to financial and narrative disclosures, protect investors' rights and interests, and impose severe penalties on those involved in disclosure violations.

Second, companies and executives should take action to improve disclosure quality and avoid information manipulation. On the one hand, information disclosures are a primary means of alleviating information asymmetry (Li, 2010). Thus, rather than disclosing information strategically manipulated to cater to investors, companies and managers should seek to improve disclosure quality and alleviate information asymmetry by disclosing financial and narrative information based on their past and present realities. On the other hand, previous studies show that executives can achieve specific goals through information manipulation (Huang et al., 2014; Kong, 2018). Furthermore, our analysis indicates that executives can manipulate intonations to cater to irrational investors. However, the information advantages of managers are temporary. The appearance of new information may enable investors to discern executives' information manipulation or result in additional negative consequences. Therefore, companies and executives should avoid information manipulation and misleading disclosures.

## Limitations and future research

Despite its theoretical and practical contributions, this study still has several shortcomings. The first is that the investor sentiment variable we adopt is a market-level investor indicator designed to alleviate quid endogeneity between investor sentiment and executive tone. However, some studies construct firm-level indicators of investor sentiment (e.g., Mahmoudi, 2022). Therefore, future studies should consider ways to research the relationship between investor sentiment and executive tone by adopting firm-level sentiment indicators without endogeneity problems.

Second, our research finds that executives adopt intonation tactics that involve using more positive words relative to negative words to cater to the sentiment of overly optimistic investors. In addition, managers may adopt manipulated tones in response to irrational investors. However, investors' emotions are not always overly optimistic (Byun et al., 2021). How executives change the intonations of their disclosures and tactics in response to investor sentiment in situations where investors are overly pessimistic remains an open question. Therefore, future studies should research the relationship between investor sentiment and executive tone in an overly pessimistic investor context.

Finally, executives tend to adopt different intonation tactics, including manipulated and real intonations, in response to investor sentiment. However, different intonations in disclosures may result in varied economic consequences. Thus, future studies should investigate the consequences of the different intonation catering tactics executives deploy.

## Conclusion

Adopting the Catering Theory perspective, this study investigates the association between investor sentiment and executive tone by empirically analyzing 20,328 samples from the Chinese equity market. We find that executives tend to adopt a more positive intonation to cater to irrational market-level investor sentiment and may change their catering tactics in response to stock price and trading volume pressures on executives and firm-level investors' expectations and preferences. In addition, we show that rational investors can change the intonations of their disclosures and adopt different intonation tactics to respond to investor sentiment. We hope that this study will provide a framework for additional research into these subjects.

## Data availability statement

The original contributions presented in the study are included in the article/supplementary material, further inquiries can be directed to the corresponding author.

## Author contributions

JQ and NY: conceptualization, methodology, and writing. NY: data collection and formal analysis. JQ: supervision and review. The published version of the manuscript has been read and approved by both authors.

## Funding

This research was funded by the 2022 School-level Projects of Guizhou University of Finance and Economics, Grant Number 2022KYYB03.

## Conflict of interest

The authors declare that the research was conducted in the absence of any commercial or financial relationships that could be construed as a potential conflict of interest.

## Publisher's note

All claims expressed in this article are solely those of the authors and do not necessarily represent those of their affiliated organizations, or those of the publisher, the editors and the reviewers. Any product that may be evaluated in this article, or claim that may be made by its manufacturer, is not guaranteed or endorsed by the publisher.
